# ERK5 and Cell Proliferation: Nuclear Localization Is What Matters

**DOI:** 10.3389/fcell.2016.00105

**Published:** 2016-09-22

**Authors:** Nestor Gomez, Tatiana Erazo, Jose M. Lizcano

**Affiliations:** Protein Kinases and Signal Transduction Laboratory, Institut de Neurociencies and Departament de Bioquimica i Biologia Molecular, Facultat de Medicina, Universitat Autonoma de BarcelonaBarcelona, Spain

**Keywords:** MAP kinase, ERK5, nuclear translocation, transcriptional co-activator, cell proliferation, cancer, Hsp90, Cdc37

## Abstract

ERK5, the last MAP kinase family member discovered, is activated by the upstream kinase MEK5 in response to growth factors and stress stimulation. MEK5-ERK5 pathway has been associated to different cellular processes, playing a crucial role in cell proliferation in normal and cancer cells by mechanisms that are both dependent and independent of its kinase activity. Thus, nuclear ERK5 activates transcription factors by either direct phosphorylation or acting as co-activator thanks to a unique transcriptional activation TAD domain located at its C-terminal tail. Consequently, ERK5 has been proposed as an interesting target to tackle different cancers, and either inhibitors of ERK5 activity or silencing the protein have shown antiproliferative activity in cancer cells and to block tumor growth in animal models. Here, we review the different mechanisms involved in ERK5 nuclear translocation and their consequences. Inactive ERK5 resides in the cytosol, forming a complex with Hsp90-Cdc37 superchaperone. In a canonical mechanism, MEK5-dependent activation results in ERK5 C-terminal autophosphorylation, Hsp90 dissociation, and nuclear translocation. This mechanism integrates signals such as growth factors and stresses that activate the MEK5-ERK5 pathway. Importantly, two other mechanisms, MEK5-independent, have been recently described. These mechanisms allow nuclear shuttling of kinase-inactive forms of ERK5. Although lacking kinase activity, these forms activate transcription by interacting with transcription factors through the TAD domain. Both mechanisms also require Hsp90 dissociation previous to nuclear translocation. One mechanism involves phosphorylation of the C-terminal tail of ERK5 by kinases that are activated during mitosis, such as Cyclin-dependent kinase-1. The second mechanism involves overexpression of chaperone Cdc37, an oncogene that is overexpressed in cancers such as prostate adenocarcinoma, where it collaborates with ERK5 to promote cell proliferation. Although some ERK5 kinase inhibitors have shown antiproliferative activity it is likely that those tumors expressing kinase-inactive nuclear ERK5 will not respond to these inhibitors.

## Introduction

Extracellular signal-re gulated kinase 5 (ERK5, also named big MAP kinase 1, Bmk1) is a member of the Mitogen-activated protein kinases (MAPKs). ERK5 is ubiquitously expressed throughout all mammalian tissues and cell lines (Lee et al., 1995; Zhou et al., 1995; Buschbeck and Ullrich, 2005), where it is activated in response to several growth factors and oxidative and hyperosmotic stress (Kato et al., 2000). ERK5 is twice the size of the classical MAPKs (816 aa for human ERK5), containing an N-terminal kinase domain (aa 78–406) and a unique C-terminal tail (aa 410–816) with no homology to any other protein, which has an autoinhibitory function (Buschbeck and Ullrich, 2005). This C-terminal tail contains a myocyte enhancer factor 2 MEF2-interacting region (aa 440–501, Kasler et al., 2000), a bipartite nuclear localization signal NLS (aa 505–539), and a transcriptional activation domain (TAD, aa 664–789, Kasler et al., 2000), which associates with and activates several transcription factors (Morimoto et al., 2007; Woo et al., 2010). Activation of ERK5 requires dual phosphorylation of a Threonine and Tyrosine residues within a TEY motif in the activation loop of the kinase domain (Mody et al., 2003). MEK5 is the only kinase that activates ERK5, and it has a unique specificity for ERK5, not phosphorylating any other MAPKs: MEK5 knockout mice dye at early stages of embryo development, showing similar defects in cardiac development and angiogenesis as those reported for ERK5 knockout mice (Wang et al., 2005).

## Role of ERK5 pathway in normal and cancer cell proliferation

ERK5 plays a crucial role in cell proliferation. First evidences reported that ERK5 activation is required for EGF-dependent proliferation in HeLa cells (Kato et al., 1998), by inducing transcription of *c-jun* (an essential component in cell proliferation) through the transcriptional activation of MEF2C (Kato et al., 1997). Since then, several authors have shown activation of ERK5 in response to other mitogenic factors, such as Nerve growth factor (NGF, Shao et al., 2002), Granulocyte colony-stimulating factor (G-CSF, Dong et al., 2001), Fibroblast growth factor (FGF, Kesavan et al., 2004), or Platelet-derived growth factor (PDGF, Rovida et al., 2008).

ERK5 regulates cell cycle progression, being necessary for G1/S transition. In this regard, ERK5 inhibition prevents cells from entering the S phase of the cell cycle (Kato et al., 1998) by stabilizing the cyclin-dependent protein kinase (CDK) inhibitors p21 and p27 (Perez-Madrigal et al., 2012). In human breast cancer MDA-MB-231 cells, activation of ERK5 promotes c-Myc-dependent transcriptional activation of miR-17-5p and miR-20a, resulting in blockade of p21 mRNA translation (Perez-Madrigal et al., 2012). ERK5 also mediates in G1/S transition by regulating expression of cyclin D1. Activation of MEK5/ERK5 pathway induces transcription of Cyclin D1, resulting in cell cycle progression in G1. Conversely, ERK5 inhibition diminishes serum-induced Cyclin D1 protein levels (Mulloy et al., 2003). Additionally, ERK5 is also implicated in G2/M transition. ERK5 is activated at G2/M, it is required for timely mitotic entry, and constitutively active ERK5 increases the mitotic index (Cude et al., 2007; Girio et al., 2007). The mitotic entry induced by ERK5 depends on the activation of the transcription factor NF-kB, which upregulates mitosis-promoting genes such as cyclins B1 and B2, and cdc25B (Cude et al., 2007). During mitosis, active ERK5 prevents caspase activation by binding and inactivating the pro-apoptotic protein Bim, suggesting that active ERK5 contributes to cell survival in mitosis (Girio et al., 2007). The role of ERK5 in controlling cell survival and differentiation, as well as angiogenesis, has been already covered in excellent reviews (Wang and Tournier, 2006; Drew et al., 2012; Lochhead et al., 2012; Nithianandarajah-Jones et al., 2012).

During the last years, different laboratories have shown that the MEK5-ERK5 pathway plays a key role in cancer cell proliferation. For instance, overexpression of either MEK5 or ERK5 in prostate adenocarcinoma PC-3 cells results in increased proliferation index (McCracken et al., 2008; Erazo et al., 2013). Consequently, ERK5 kinase inhibitors (such as the XMD8-92 compound) or ERK5 silencing show antiproliferative activity in different cancer cell lines and block tumor growth in animal models (human tumor xenografts). Table 1 summarizes the different human cancers where it has been reported an effect of ERK5 silencing/inhibition on cell proliferation and/or tumor growth. Importantly, there are increasing evidences pointing to an important role of nuclear ERK5 in cancer, both *in vitro* (cell lines) and *in vivo* (mouse models). For instance, there is a strong correlation between nuclear ERK5 and poor prognosis in prostate cancer patients. Expression of nuclear ERK5 is upregulated in prostate cancers showing high-grade Gleason and bone metastasis (McCracken et al., 2008; Clape et al., 2009; Ramsay et al., 2011; Ahmad et al., 2013). However, and as explained below, ERK5 can promote cell proliferation independently of its kinase activity, acting as a moonlighting protein. For instance, hepatocellular carcinoma (HCC) tumors show increased nuclear ERK5, which does not correlate with an increase on ERK5 kinase activity (Rovida et al., 2015). Also, ERK5 localizes at the nucleus of CLB-BAR and CLB-GE human neuroblastoma cell lines, even in the presence of the ERK5 inhibitor XMD8-92 (Umapathy et al., 2014). These findings suggest that nuclear ERK5 expression, instead of ERK5 phosphorylation, might be used as prognostic biomarker of some cancers.

**Table 1:** **Effect of ERK5 silencing or inhibition on cancer cell proliferation and tumor growth**.

**Type of cancer**	**Target strategy**	**Effect**	**References**
Leukemia	Silencing	shERK5 blocks tumor formation	Garaude et al., 2006; Charni et al., 2009
Lung carcinoma	XMD8-92 inhibition	XMD8-92 blocks tumor proliferation and angiogenesis in LL/2 and A59 xenograft models	Yang et al., 2010
Prostate cancer	Silencing	ERK5 silencing inhibits PC-3 cell proliferation and invasion. ERK5 overexpression induces more metastatic lesions in an orthotopic prostate model	Ramsay et al., 2011
Osteosarcoma	Silencing	ERK5 silencing reduces the number of invading cells	Kim et al., 2012
Malignant mesothelioma	Silencing	Injection of shERK5 malignant mesothelioma cell lines into SCID mice shows reduction in tumor growth	Shukla et al., 2013
Clear cell renal carcinoma	Silencing	ERK5 knockdown reduces proliferation and migration of 769-P and 786-O cells	Arias-Gonzalez et al., 2013
Hepatocellular carcinoma (HCC)	XMD8-92 inhibition Silencing	ERK5 inhibition or silencing inhibits EGF-induced cell migration. XMD8-92 reduces size of HCC xenograft tumors	Rovida et al., 2015
Triple negative breast cancer	XMD8-92 inhibition	XMD8-92 synergizes with chemotherapy (docetaxel + doxorubicin) or Hsp90 inhibitors to reduce growth of TNBC xenograft tumors	Al-Ejeh et al., 2014
Triple negative breast cancer	Silencing	ERK5 knockdown blocks TNBC cell proliferation	Ortiz-Ruiz et al., 2014
Pancreatic ductal adenocarcinoma	XMD8-92 inhibition	XMD8-92 inhibits growth of AsPC-1 tumor xenografts	Sureban et al., 2014
Neuroblastoma	XMD8-92 inhibition	XMD8-92 reduces growth of CLB-BAR and CLB-GE tumor xenografts. Also, synergizes with crizotinib to reduce growth of these tumors	Umapathy et al., 2014
Skin cancer	XMD8-92. ERK5 conditional KO in epidermis	XMD8-92 blocks skin tumor development and potentiates doxorubicin action. ERK5-KO keratinocyte show impair inflammation-driven tumorigenesis	Finegan et al., 2015

## ERK5 nuclear substrates

The best characterized ERK5 substrates are nuclear transcriptional factors, whereas very few ERK5 cytosolic substrates have been characterized so far. Although ERK5 silencing affects the phosphorylation state of cytosolic proteins such as Akt and p90RSK kinases or the pro-apoptotic protein BAD, this is controversial since a direct ERK5 phosphorylation of these proteins has not been shown. For instance, Ranganathan et al. described that ERK5 phosphorylates p90RSK *in vitro* (Ranganathan et al., 2006), but other authors have shown that the MEK1/2 inhibitor PD184352 blocks p90RSK activation in response to EGF, at concentrations that do not block ERK5 activity (Mody et al., 2001). The use of the new synthesized specific ERK5 inhibitors—such as the XMD8-92 compound—will help to address these controversies.

ERK5 phosphorylates the transcription factor Sap1, a member of ternary complex factors (TCFs). ERK5-mediated Sap1 phosphorylation activates transcription through the Serum Response Element (SRE), which induces the expression of c-Fos (Kamakura et al., 1999). Although a direct phosphorylation by ERK5 has not been shown, co-expression of ERK5 and catalytically active MEK5 in COS-7 cells induces phosphorylation and stabilization of c-Fos and Fra-1 transcription factors (Terasawa et al., 2003). Activation of the ERK5 pathway results in phosphorylation of few Ser/Thr residues in c-Fos and Fra-1, generating more stable proteins and enhanced transactivation activity of these factors (Terasawa et al., 2003).

The three members of the MEF2 (myocyte enhancer factor-2) family of transcription factors MEF2A, MEF2C, and MEF2D are the best characterized ERK5 substrates. MEF2 proteins regulate cell differentiation in myocytes and neurons (Potthoff and Olson, 2007), and act as a nodal point for *stress-response* in adult tissues (Kim et al., 2008). An interaction of MEF2C with ERK5 has been shown in two hybrid and co-immunoprecipitation assays. To our knowledge, MEF2C is the only nuclear protein whose interaction with ERK5 has been mapped. MEF2C interacts with a region of the C-terminal tail of ERK5 (aa 440–501) through its N-terminal end (Yang et al., 1998; Kasler et al., 2000). More importantly, activation of ERK5 pathway by either serum or EGF stimulates the transactivation activity of the MEF2A, MEF2C, and MEF2D transcription factors. Furthermore, ERK5 seems to be determinant for EGF-stimulated activation of MEF2A and MEF2D, since expression of ERK5 dominant negative mutant abolishes this activation (Kato et al., 2000). ERK5 phosphorylates different sites in MEF2A, MEF2C, and MEF2D proteins, however many of these residues are conserved in all three members, suggesting the existence of additional structural determinants to achieve recognition of the phosphorylated sites. Thus, ERK5 phosphorylates Thr312, Thr319, and S355 in MEF2A, Ser386/7 in MEF2C, and Ser179 and in MEF2D, and mutation to alanine of these residues abrogates MEF2 transcriptional activity (Kato et al., 1997, 2000).

The promyelocytic leukemia protein (PML) is a transcription factor that acts as a tumor suppressor, inhibiting proliferation and inducing cellular senescence and apoptosis through activation of the CDK inhibitor p21 (Bernardi and Pandolfi, 2007). Yang et al. have shown that ERK5 interacts with PML at the nuclear bodies in cancer cells, and inhibits its tumor suppressor activity by phosphorylating PML protein at Ser403 and Thr409 (Yang et al., 2010). ERK5-mediated phosphorylation impairs PML-dependent activation of p21, through disrupting PML-MDM2 interaction, and downregulating expression of the p53 tumor suppressor (Yang et al., 2013).

## Mechanisms involved in ERK5 nuclear translocation

ERK5 acts as a transcriptional co-activator, regulating MEF2C, AP-1, and c-Fos transcriptional activities in the nucleus. Therefore, translocation of ERK5 to nucleus is essential to regulate ERK5-mediated gene transcription. ERK5 is a big protein (110 kDa) so it cannot enter the nucleus by passive diffusion through the nuclear pores, as described for small proteins.

Recently, we have shown that inactive ERK5 binds the cytoplasmatic chaperone Hsp90 and the co-chaperone cell division-cycle 37 (Cdc37), which helps Hsp90 in the stabilization of ERK5 (Erazo et al., 2013). Cdc37 is the co-chaperone that specifically promotes association of Hsp90 with many protein kinases (Smith and Workman, 2009). This trimeric complex, ERK5-Hsp90-Cdc37, not only stabilizes inactive ERK5, but also keeps ERK5 in a suitable conformation for MEK5 recognition and activation (Erazo et al., 2013).

In basal conditions, ERK5 binds the cytoplasmatic chaperone Hsp90, which serves as a cytosolic anchor for ERK5 (Erazo et al., 2013). This inactive ERK5 adopts a closed conformation where the C-terminal tail interacts with the kinase domain and the NLS motif is hidden and not available for the nuclear transport (reviewed in Kondoh et al., 2006). Nuclear shuttling of ERK5 requires both a conformational change to allow exposure of the NLS motif and the release of Hsp90. This mechanism is analogous to the one described for the progesterone and androgen receptors, which also requires Hsp90 dissociation for their nuclear translocation (Picard, 2006).

So far, two different mechanisms have been proposed for the ERK5 shuttling to the nucleus: one of them requires C-terminal phosphorylation, while the other does not. Once in the nucleus, ERK5 enhances gene transcription by either phosphorylating transcription factors, or by interacting with these factors through the transactivation TAD domain located at the C-terminal. Strikingly, ERK5 does not require kinase activity to interact with and activate transcription factors and therefore, forms of nuclear ERK5 devoid of kinase activity are able to activate transcription (Diaz-Rodriguez and Pandiella, 2010; Inesta-Vaquera et al., 2010; Erazo et al., 2013). Kinase-independent nuclear functions have been also proposed for other MAP kinases that lack a TAD domain. For instance, ERK2 promotes cell cycle entry by disrupting retinoblastoma-lamin A complexes in a kinase-independent fashion (Rodriguez et al., 2010), and also binds DNA acting as a transcriptional repressor for interferon gamma-induced genes (Hu et al., 2009). It would be interesting to study if ERK5 can also bind DNA.

### Nuclear translocation dependent of ERK5 C-Terminal phosphorylation

**Figure 1:**
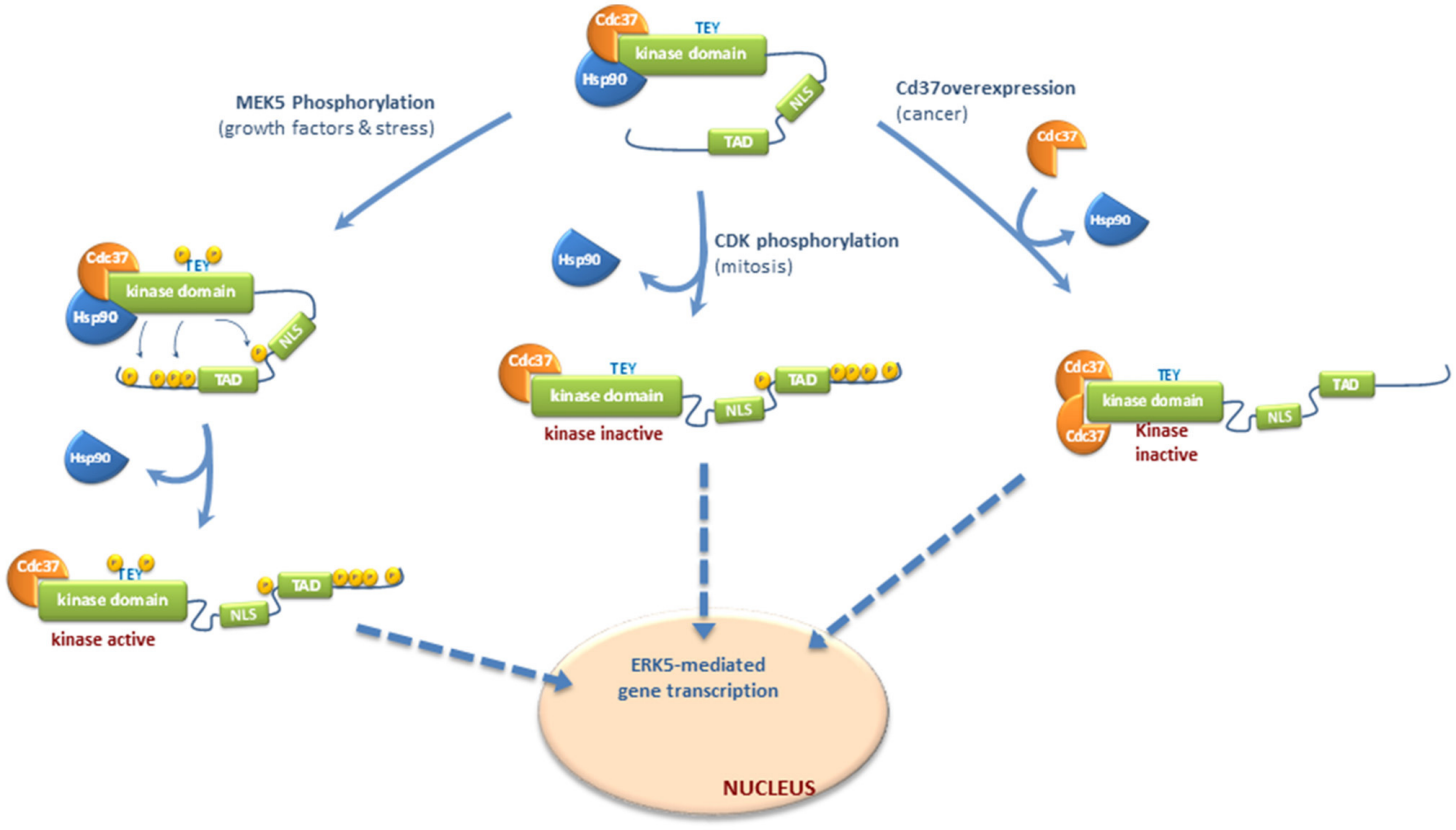
**Molecular mechanisms controlling nucleocytoplasmatic transport of ERK5**. Different mechanisms have been proposed for ERK5 shuttling to the nucleus, depending or not of C-terminal phosphorylation. Once in the nucleus, ERK5 enhances gene transcription by either phosphorylating transcription factors, or by interacting with these factors through the transactivation TAD domain located at the C-terminal. ERK5 does not require kinase activity to interact with and activate transcription factors and therefore, forms of nuclear ERK5 devoid of kinase activity are able to activate transcription. Inactive ERK5 binds the cytoplasmatic chaperone Hsp90 and co-chaperone Cdc37, which helps Hsp90 in the stabilization of ERK5 (Erazo et al., 2013). In basal conditions, Hsp90 serves as a cytosolic anchor for ERK5, and inactive ERK5 adopts a closed conformation where the NLS motif is hidden and not available for the nuclear transport. Nuclear shuttling of ERK5 requires both a conformational change to allow exposure of the NLS motif and the release of Hsp90 (Erazo et al., 2013). In response to growth factors stimulation or different stresses, activated MEK5 phosphorylates and activates ERK5. Then, active ERK5 phosphorylates its C-terminal tail resulting in dissociation of the cytosolic anchor Hsp90 from ERK5-Cdc37 complex, adoption of an open conformation in which the NLS motif is exposed, and nuclear translocation. ERK5 C-terminal tail can also be phosphorylated by other kinases, such as Cyclin-dependent kinase-1 (CDK1) during mitosis. This phosphorylation is not MEK5-dependent but induce the release of Hsp90 and nuclear translocation of a kinase-inactive form of ERK5 which retains its transcriptional activity (Diaz-Rodriguez and Pandiella, 2010; Inesta-Vaquera et al., 2010). Finally, overexpression of Cdc37 (reported to happen in some cancers) induces the release of Hsp90 and nuclear shuttling of a kinase-inactive but transcriptionally active form of ERK5 (Erazo et al., 2013).

In response to EGF stimulation or different stresses MEK5 becomes activated, which in turn activates ERK5 by dual phosphorylation of the TEY motif. Then, active ERK5 phosphorylates its C-terminal tail resulting in a sequence of events that include: (1) dissociation of the cytosolic anchor Hsp90 from ERK5-Cdc37 complex; (2) adoption of a conformation in which the NLS motif is exposed; and (3) nuclear translocation (Figure 1).

ERK5 autophosphorylates several Thr/Ser residues within its C-terminal region. Mody et al., using purified recombinant active ERK5 and mass-spectrometry analysis, first identified five autophosphorylation sites at the C-terminal region: Ser421, Ser433, Ser496, Ser731, and Thr733 (Mody et al., 2003). Another laboratory also reported autophosphorylation in residues Ser760, Ser764, and Ser766, in addition to Thr733 (Morimoto et al., 2007). Inhibition of ERK5 autophosphorylation prevents the release of Hsp90 and nuclear entry (Erazo et al., 2013), reflecting that C-terminal tail autophosphorylation plays a critical role in ERK5 nuclear shuttling in response to MEK5 stimulation. Consequently, mutant forms of ERK5 in which the autophosphorylated residues at the C-terminal were mutated to alanine show cytoplasmatic localization and constitutive association to Hsp90 (Erazo et al., 2013), whereas the mutant in which these residues were mutated to glutamic acid does not bind Hsp90 (Erazo et al., 2013) and shows nuclear localization (Morimoto et al., 2007; Diaz-Rodriguez and Pandiella, 2010). Therefore, autophosphorylation of the C-terminal tail induces the release of Hsp90 from the ERK5-Cdc37 complex, a step essential previous nuclear translocation.

ERK5 C-terminal tail can also be phosphorylated by other kinases. During mitosis, ERK5 is phosphorylated at residues Ser567, Ser720, Ser731, Thr733, Ser753, and Ser830 (Diaz-Rodriguez and Pandiella, 2010; Inesta-Vaquera et al., 2010). These phosphorylations are not MEK5-dependent but induce the nuclear translocation of a kinase-inactive form of ERK5 which retains its transcriptional activity. These events might represent a second pathway controlling ERK5 C-terminal phosphorylation, which is activated in mitotic cells and involves kinase activities distinct from MEK5. Cyclin-dependent kinase-1 (CDK1) might well phosphorylate these sites, since either roscovitine or the RO3306 inhibitor reverse mitotic phosphorylation of ERK5 (Diaz-Rodriguez and Pandiella, 2010; Inesta-Vaquera et al., 2010).

All in all, C-terminal phosphorylation integrates different signals that converge in ERK5 nuclear shuttling and activation of transcription. ERK5 C-term autophosphorylation would represent a MEK5-dependent mechanism that integrate signals such as growth factors (EGF) and oxidative and osmotic stresses that activate MEK5-ERK5 pathway. On the other hand, C-terminal phosphorylation by other kinases (such as CDK1 during mitosis) represents a mechanism of nuclear translocation that does not require ERK5 kinase activity, but results in ERK5-mediated activation of transcription.

### Nuclear translocation independent of ERK5 C-Terminal phosphorylation

We have described a new mechanism for ERK5 nuclear translocation which is independent of C-terminal phosphorylation. Expression of high levels of Cdc37 induces the release of Hsp90 and the nuclear shuttling of a kinase-inactive form of ERK5 that retains its transcriptional activity (Erazo et al., 2013). This mechanism does not involve ERK5 activation or C-terminal phosphorylation by other kinases; overexpression of Cdc37 induced nuclear translocation and ERK5-mediated gene transcription in the presence of the specific inhibitor XMD8-92 or in MEK5 KO cells (Erazo et al., 2013).

The relevance of this new mechanism relays on the fact that Cdc37 acts as an oncogene, stabilizing other oncogenes that are mutated or overexpressed in cancer cells such as Akt, Her-2, or BRAF (Smith and Workman, 2009). Overexpression of Cdc37 has been observed in prostate adenocarcinoma, where it collaborates with c-Myc and cyclin D1 in the transformation of this tumor (Stepanova et al., 2000; Gray et al., 2007). Remarkably, *in vitro* evidences suggest that Cdc37 collaborates with ERK5 to promote proliferation of PC3 prostatic adenocarcinoma cells (Erazo et al., 2013). Cdc37 is also overexpressed in acute myelocytic leukemia and multiple myeloma (Casas et al., 2003; Katayama et al., 2004). It would be interesting to explore in these cancer cells if ERK5 shows constitutive nuclear localization and also collaborates with Cdc37 to promote cell proliferation. If so, and given the fact that Cdc37 induces nuclear shuttling of a kinase-inactive form of ERK5, we predict that these cancers will not respond to ERK5 inhibitors.

## Perspectives

During the last years, efforts of many laboratories have led to delineate the importance of ERK5 in controlling cell proliferation in normal and cancer cells, by mechanisms that are both dependent and independent of its kinase activity: nuclear ERK5 activates transcription factors by either direct phosphorylation or acting as co-activator thanks to a unique transcriptional activation domain located at its C-terminal tail. Consequently, ERK5 has been proposed as an interesting target to tackle different cancers, and either inhibitors of ERK5 activity or silencing the protein have shown antiproliferative activity in cancer cells and to block tumor growth in animal models. However, and as we have seen above, ERK5 kinase inhibitors such as XMD8-92 might not be useful in cancers showing kinase-inactive nuclear ERK5. On the other hand, the anticancer activity of ERK5 inhibitors should be carefully interpreted. It has been recently reported that several classes of kinase inhibitors can strongly inhibit the bromodomain-containing protein-4 Brd4, a general transcription co-activator (Ciceri et al., 2014). This is the case for XMD8-92, which inhibits Brd4 and ERK5 with similar potency (Lin et al., 2016), and therefore some of the antitumor effects of this compound could be mediated by the Brd4-inhibiting activity. A new generation of specific ERK5 inhibitors are required to clarify the exact role of ERK5 in cancer cell growth.

There are still many open questions that remain to be addressed in order to describe the precise mechanism involved in ERK5 nuclear shuttling. For instance, is CDK1 the only kinase that phosphorylates ERK5 C-terminal tail? Does Cdc37 overexpression mimic a physiological mechanism that allows nuclear shuttling of inactive ERK5? Are other post-translational modifications, such as SUMOylation, required for ERK5 nuclear translocation? What is the role of ERK5 phosphatases in this process? The very near future seems an exciting time to deal with these questions, and hopefully will provide clues to design new compounds with antiproliferative activity.

## Author contributions

All three authors wrote and corrected the manuscript. TE desgined Table 1. JL designed Figure 1.

### Conflict of interest statement

The authors declare that the research was conducted in the absence of any commercial or financial relationships that could be construed as a potential conflict of interest.
